# Age-related changes of the periocular morphology: a two- and three-dimensional anthropometry study in Caucasians

**DOI:** 10.1007/s00417-022-05746-y

**Published:** 2022-08-15

**Authors:** Jinhua Liu, Alexander C. Rokohl, Honglei Liu, Wanlin Fan, Senmao Li, Xiaoyi Hou, Sitong Ju, Yongwei Guo, Ludwig M. Heindl

**Affiliations:** 1 Department of Ophthalmology, Xi’an Fourth Hospital, Jiefang Road 21, 710004 Xi’an, Shaanxi China; 2 Department of Ophthalmology, Faculty of Medicine and University Hospital Cologne, University of Cologne, Kerpener Strasse 62, 50937 Cologne, Germany; 3 Department of Ophthalmology, West China Hospital, Sichuan University, Guoxue Alley 37, Wuhou District, 610041 Chengdu, Sichuan China; 4 Eye Center, Second Affiliated Hospital, Zhejiang University School of Medicine, Jiefang Road 88, 310009 Hangzhou, Zhejiang China

**Keywords:** Periocular morphology change, Age-related, Sex-related, Endocanthion, Pupil, Exocanthion

## Abstract

**Purpose:**

To determine age-and sex-related changes in periocular morphology in Caucasians using a standardized protocol.

**Methods:**

Healthy Caucasian volunteers aged 18–35 and 60–90 years old were recruited from the Department of Ophthalmology, Faculty of Medicine and University Hospital, Cologne, between October 2018 and May 2020. Volunteers with facial asymmetry, facial deformities, history of facial trauma, facial surgery, botox injection, eyelid ptosis, strabismus, or nystagmus, were excluded. Standardized three-dimensional facial photos of 68 young volunteers and 73 old volunteers were taken in this clinical practice. Position changes of endocanthion, pupil center, and exocanthion were analyzed in different age and gender groups, including palpebral fissure width (PFW): distance between endocanthions (En-En), pupil centers (Pu–Pu), exocanthions (Ex-Ex), endocanthion and nasion (En-Na), pupil center and nasion (Pu-Na), exocanthion and nasion (Ex-Na), endocanthion and pupil center (Pu-En), exocanthion and pupil center (Pu-Ex), and palpebral fissure inclination (PFI); angle of endocanthions to nasion (En-Na-En), pupils to nasion (Pu-Na-Pu), exocanthions to nasion (Ex-Na-Ex); endocanthion inclination (EnI), and exocanthion inclination (ExI).

**Results:**

PFW, En-En, Ex-Na, Pu-Ex, PFI, ExI, and Ex-Na-Ex were significantly different between the young and old groups (*p* ≤ 0.004). There were sex-related differences in PFW, Ex-Ex, En-Na, Pu-Na, Ex-Na, Pu-En, PFI, and EnI between both groups (*p* ≤ 0.041).

**Conclusion:**

The position change of the pupil is minimal relative to age; it is preferred to establish the reference plane to describe periocular changes. The endocanthion tends to move temporally and inferiorly, while the exocanthion tends to shift nasally and inferiorly with age.


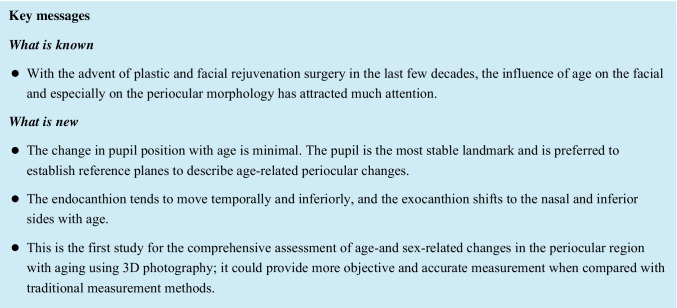


## Introduction

With the advent of plastic and facial rejuvenation surgery in the last few decades [1], the influence of age on the facial and especially on the periocular morphology has attracted much attention [1–3]. Some studies investigated how aging affects facial structures to improve the outcomes of facial rejuvenation surgeries [2, 4–12]. However, at present, comprehensive studies on how aging changes the periocular morphology in Caucasian populations are insufficient; a standardized examination protocol with defined reference landmarks is missing.

There is always a need for surgical plans for eye rejuvenation surgery to be strictly designed before the surgeries. However, as there is no standardized examination protocol and specific standards for reference, this process is currently difficult to achieve. Surgeons often need to make supervisory judgments instead of a standardized process, which might relate to the unsatisfactory results after eye rejuvenation surgeries.

Evaluation of position changes of the periocular soft tissue could draw conflicting conclusions due to the lack of reliable reference landmarks. For example, Pieter et al. [3, 13] used the mid pupil as a reference line, while other reports [4, 14–16] used either the endocanthion or exocanthion to compare the eyebrow position [17, 18]. Price et al. [19] considered that eyebrow elevation occurs with aging. However, Lambros et al. [10] found that brows descended in 29% of patients. These contrasting conclusions may be related to the displacement of the reference plane. Although different methods to assess the brow position have been described [3, 6, 9, 14, 15, 20, 21], no standardized protocol has been defined [13]. Therefore, it is important to establish a validated stable reference plane with a minimal age-related variation.

Most existing studies compared morphological changes only using two-dimensional photography or cephalograms to analyze the variables [22, 23]. However, the development of three-dimensional stereophotography holds considerable promise for quantitative and accurate assessment of soft tissue, including the periocular region [22, 24–35].

Therefore, this study aimed to investigate age- and sex-related changes in ocular morphology in a Caucasian population using two- and three-dimensional stereophotography, to identify more reliable landmarks, thus establishing a standardized examination protocol for subsequent studies and routine clinical assessment.

## Materials and methods

Healthy Caucasian volunteers aged 18–35 and 60–90 years were recruited in our study from the Department of Ophthalmology, Faculty of Medicine and University Hospital, Cologne, between October 2018 and May 2020. Volunteers with facial asymmetry, facial deformities, history of facial trauma, facial surgery, botox injection, eyelid ptosis, strabismus, or nystagmus were excluded. This study complied with the Declaration of Helsinki and its later amendments. Approval was obtained from the local institutional ethics committee (No. 17–199), and written informed consent was obtained from all participants.

### Stereophotography

Stereophotography was conducted using the VECTRA M3 3D imaging system (Canfield Scientific, Inc., Fairfield, NJ, USA). Images were captured while the volunteers looked straight forward without any expression in the Frankfort horizontal (FH) plane. The same operator took the photographs. The 2D images were converted from 3D images using Mirror software (Canfield Scientific, Inc., Fairfield, NJ, USA). The camera was calibrated before each use.

### Measurements

**Fig. 1:**
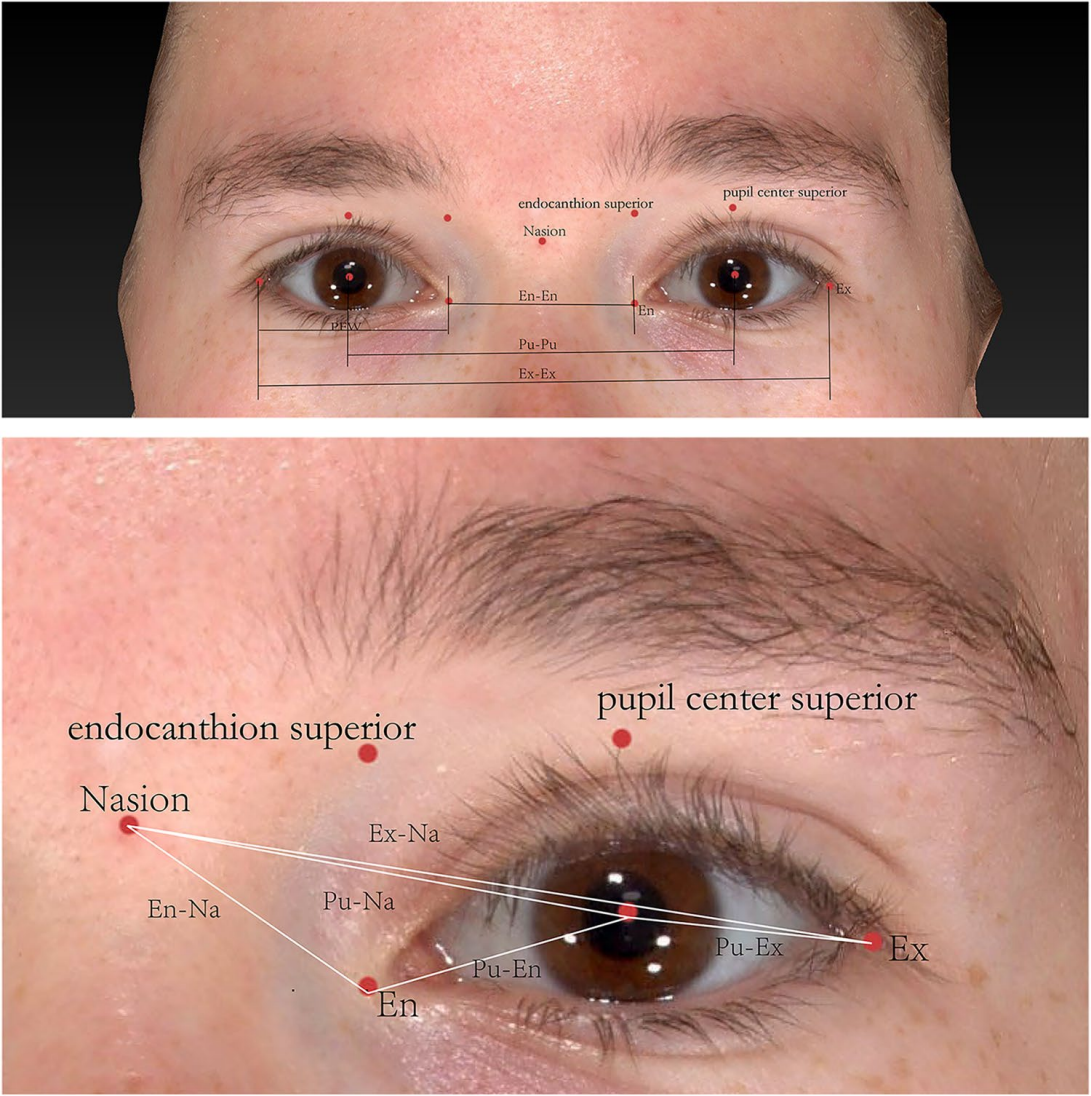
Landmarks and methods of the nine linear measurements

For each patient, landmarks including the nasion; endocanthion; exocanthion; the pupil centers; the pupil center superior landmarks (on the perpendicular line passing through the pupil center); and the endocanthion superior landmarks (on the perpendicular line passing through the endocanthion) were labeled, as shown in Fig. 1.

The following nine linear distances (Fig. 1) were measured in the 3D images: (1) palpebral fissure width; (2) distance between endocanthions (En-En); (3) distance between pupil centers (Pu–Pu); (4) distance between exocanthions (Ex-Ex); (5) distance between the endocanthion and nasion (En-Na); (6) distance between the pupil center and the nasion (Pu-Na); (7) distance between exocanthion and nasion (Ex-Na); (8) distance between the endocanthion and the homolateral pupil center (Pu-En); and (9) distance between exocanthion and the homolateral pupil center (Pu-Ex).

**Fig. 2:**
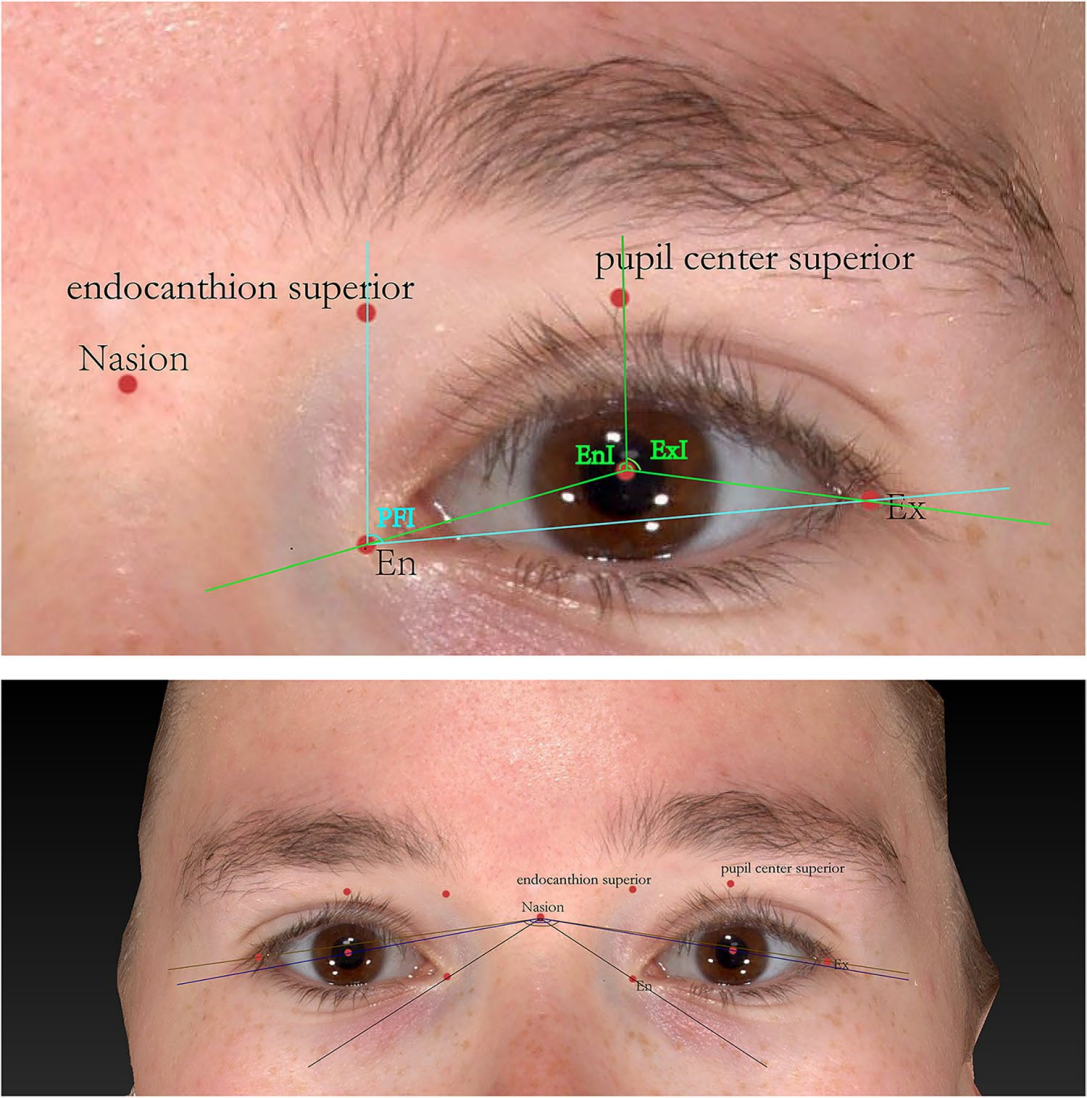
Landmarks and methods of the six angular measurements

Six angular measurements (Fig. 2) were carried out in 2D photographs: (1) The palpebral fissure inclination (PFI): the angle between the endocanthion superior landmark-endocanthion and endocanthion-exocanthion; (2) the angle of the endocanthions to nasion (En-Na-En); (3) the angle of the pupils to nasion (Pu-Na-Pu); (4) the angle of the exocanthions to nasion (Ex-Na-Ex); (5) the endocanthion inclination (EnI)—the angle between the pupil center superior landmark-pupil center line and the pupil center-endocanthion line; and (6) the exocanthion inclination (ExI)—the angle between the line of the pupil center superior landmark-pupil center and the line of pupil center-exocanthion.

### Statistical analysis

SPSS version 25.0 (IBM Corporation, Armonk, NY*,* USA*)* was used for statistical analyses. An independent sample *t* test was performed after the normal distribution was verified using the Kolmogorov–Smirnov test to analyze differences between groups. The statistical significance level was set at *p* < 0.05.

## Results

A total of 141 Caucasian volunteers, including 68 young and 73 old participants, were recruited. The age composition of each group and the differences of nine linear distance and six angular measurements between the young and old groups are shown in Table 1. Differences of measurements between different ages are shown in Table 2.

**Table 1:** Age composition and measurements differences between the young and old groups

Age range (years old)	Male	Female	Total
Young group	20–34	19–33	19–34
Old group	61–88	63–88	61–88
Mean ± SD (years old)	Male	Female	Total
Young group	27.42 ± 3.64	26.13 ± 3.9	26.81 ± 3.86
18–20	20.00 ± 0.00	19.00 ± 0.00	19.33 ± 0.47
21–30	26.36 ± 2.59	25.48 ± 2.89	25.94 ± 2.77
31–35	32.71 ± 0.88	32.67 ± 0.47	32.70 ± 0.78
Old group	77.03 ± 7.29	77.02 ± 7.00	77.03 ± 7.15
60–70	65.50 ± 3.69	65.33 ± 3.30	65.42 ± 3.50
70–80	74.25 ± 2.63	75.53 ± 2.90	74.87 ± 2.84
80–90	84.13 ± 2.52	83.64 ± 2.02	83.90 ± 2.31
Linear distance	Young group	Old group	*p* value
PFW	30.856 ± 2.052	28.761 ± 2.228	< 0.001**
En-En	31.346 ± 2.769	33.306 ± 3.267	< 0.001**
Pu–Pu	62.967 ± 3.387	64.140 ± 3.570	0.054
Ex-Ex	91.485 ± 4.463	90.180 ± 5.150	0.114
En-Na	23.592 ± 2.183	24.035 ± 2.326	0.253
Pu-Na	35.291 ± 2.218	35.829 ± 2.879	0.218
Ex-Na	51.479 ± 3.032	49.846 ± 3.563	0.004*
Pu-En	16.101 ± 1.258	16.088 ± 1.573	0.958
Pu-Ex	16.258 ± 1.585	14.149 ± 1.388	< 0.001**
Angles	Young group	Old group	*p* value
PFI	88.193 ± 2.684	90.218 ± 3.219	< 0.001**
EnI	102.324 ± 4.404	101.622 ± 4.368	0.685
ExI	99.875 ± 3.576	104.144 ± 5.187	0.234
En-Na-En	119.025 ± 16.618	117.921 ± 15.433	0.003*
Pu-Na-Pu	158.560 ± 12.517	156.156 ± 11.207	0.348
Ex-Na-Ex	159.400 ± 9.723	154.640 ± 9.123	< 0.001**

*PFW*, the palpebral fissure width; *En-En*, the distance between two endocanthions; *Pu–Pu*, the distance between two pupil centers; *Ex-Ex*, the distance between two exocanthions; *En-Na*, the distance between endocanthion and nasion; *Pu-Na*, the distance between pupil center and the nasion; *Ex-Na*, the distance between exocanthion and nasion; *Pu-En*, the distance between endocanthion and homolateral pupil center; *Pu-Ex*, the distance between exocanthion and homolateral pupil center; *PFI* stands for the palpebral fissure inclination, calculated by the angle between two lines: the endocanthion superior landmark-endocanthion line and endocanthion-exocanthion line. *EnI*; the endocanthion inclination; *ExI*, the exocanthion inclination; *En-Na-En*, the angle of the endocanthions to nasion. *Pu-Na-Pu*, the angle of the pupils to nasion; *Ex-Na-Ex*, the angle of the exocanthions to nasion

**Table 2:** Differences of the nine linear distance and six angular measurements between different ages

	F						M					
	YF-G1	YF-G2	YF-G3	G1-G2	G1-G3	G2-G3	YM-G1	YM-G2	YM-G3	G1-G2	G1-G3	G2-G3
Linear distance												
PFW	0.232	< 0.001**	< 0.001**	0.287	0.161	0.385	0.031*	0.001*	< 0.001**	0.980	0.324	0.107
En-En	0.178	0.063	< 0.001**	0.906	0.215	0.045	0.189	0.147	0.066	0.732	0.955	0.625
Pu–Pu	0.084	0.147	0.035*	0.522	0.885	0.521	0.903	0.261	0.990	0.419	0.911	0.364
Ex-Ex	0.535	0.101	0.456	0.129	0.353	0.478	0.87	0.479	0.174	0.794	0.475	0.44
En-Na	0.420	0.990	0.031*	0.487	0.024*	0.077	0.105	0.402	0.766	0.297	0.324	0.734
Pu-Na	0.884	0.830	0.066	0.813	0.316	0.137	0.133	0.450	0.558	0.394	0.650	0.900
Ex-Na	0.313	0.009*	0.243	0.431	0.842	0.174	0.543	0.110	0.087	0.681	0.481	0.489
Pu-En	0.800	0.632	0.099	0.935	0.293	0.132	0.588	0.466	0.651	0.923	0.507	0.324
Pu-Ex	0.074	< 0.001**	< 0.001**	0.217	0.352	0.812	0.002*	0.001*	< 0.001**	0.402	0.49	0.042
Angles												
PFI	0.843	0.202	0.002*	0.340	0.020*	0.419	0.440	0.017*	< 0.001**	0.922	0.505	0.120
En-Na-En	0.401	0.580	0.442	0.224	0.689	0.207	0.047*	0.946	0.550	0.054	0.147	0.544
Pu-Na-Pu	0.247	0.475	0.774	0.115	0.101	0.667	0.027*	0.596	0.345	0.057	0.085	0.673
Ex-Na-Ex	0.341	0.275	0.336	0.114	0.084	0.707	0.012*	0.270	0.017*	0.200	0.436	0.173
EnI	0.183	0.902	0.249	0.215	0.018*	0.388	0.668	0.119	0.700	0.598	0.871	0.299
ExI	0.037*	0.003*	0.001*	0.488	0.650	0.818	0.510	0.055	< 0.001**	0.923	0.156	0.003*

*F*, female; *M*, male; *Y*, young volunteer;. *YF*. young females; *YM*, young males; *G1*, 60–70 age subgroup; *G2*, 71–80 age subgroup; *G3*, 81–90 age subgroup; *PFW*, the palpebral fissure width; *En-En*, the distance between two endocanthions; *Pu–Pu*, the distance between two pupil centers; *Ex-Ex*, the distance between two exocanthions; *En-Na*, the distance between endocanthion and nasion; *Pu-Na*, the distance between pupil center and the nasion; *Ex-Na*, the distance between exocanthion and nasion; *Pu-En*, the distance between endocanthion and homolateral pupil center; *Pu-Ex*, the distance between exocanthion and homolateral pupil center; *PFI* stands for the palpebral fissure inclination, calculated by the angle between two lines: the endocanthion superior landmark-endocanthion line and endocanthion-exocanthion line. *EnI*, the endocanthion inclination; *ExI*, the exocanthion inclination; *En-Na-En*, the angle of the endocanthions to nasion; *Pu-Na-Pu*, the angle of the pupils to nasion; *Ex-Na-Ex*, the angle of the exocanthions to nasion. *p ≤ 0.05; **p ≤ 0.001

There were differences in PFW between young females and old females aged 70–80 and 80–90 years (*p* < 0.001), while there were differences between young men and all subgroups of old men (*p* = 0.031, 0.001, and < 0.001, respectively). Sex-related differences were observed in both the young and old groups (*p* = 0.001 and 0.005, respectively).

Differences in the nine linear distances and six angular measurements between different sexes are shown in Table 3. There were sex-related differences in the PFI in both the young and old groups (*p* = 0.010 and 0.001, respectively). There was a difference between females in the young group and those aged 80–90 years in the old group (*p* = 0.002), while males in the young group showed differences with those aged above 70 (*p* < 0.05).

**Table 3:** Differences of the nine linear distance and six angular measurements between different genders

	OF-OM			Old	Young
	G1-G1	G2-G2	G3-G3	OF-OM	YF-YM
Linear distance					
PFW	0.622	0.003*	0.135	0.001*	0.005*
En-En	0.59	0.413	0.681	0.175	0.683
Pu–Pu	0.99	0.051	0.723	0.001*	0.113
Ex-Ex	0.514	0.004*	0.247	0.001*	0.005*
En-Na	0.001*	0.003*	0.456	< 0.001**	< 0.001**
Pu-Na	0.012*	0.001*	0.168	< 0.001**	< 0.001**
Ex-Na	0.06	0.000**	0.119	< 0.001**	< 0.001**
Pu-En	0.439	0.037*	0.014*	0.003*	0.001*
Pu-Ex	0.926	0.003*	0.439	0.196	0.014*
Angles					
PFI	0.136	0.105	0.020*	0.01*	0.001*
En-Na-En	0.035*	0.745	0.112	0.707	0.078
Pu-Na-Pu	0.015*	0.814	0.131	0.496	0.031*
Ex-Na-Ex	0.024*	0.943	0.073	0.199	0.021*
EnI	0.054	< 0.001**	0.426	0.041*	0.002*
ExI	0.958	0.639	0.007*	0.117	0.264

*F*, female; *M*, male. *Y*:,young volunteers; *O*, old volunteers; *YF*, young females; *YM*, young males; *OF*, old females; *OM*, old males; *G1*, 60–70 age subgroup; *G2*, 71–80 age subgroup; *G3*, 81–90 age subgroup; *PFW*, the palpebral fissure width; *En-En*, the distance between two endocanthions; *Pu–Pu*, the distance between two pupil centers; *Ex-Ex*, the distance between two exocanthions; *En-Na*, the distance between endocanthion and nasion; *Pu-Na*, the distance between pupil center and the nasion; *Ex-Na*, the distance between exocanthion and nasion; *Pu-En*, the distance between endocanthion and homolateral pupil center; *Pu-Ex*, the distance between exocanthion and homolateral pupil center; *PFI* stands for the palpebral fissure inclination, calculated by the angle between two lines: the endocanthion superior landmark-endocanthion line and endocanthion-exocanthion line. *EnI*, the endocanthion inclination; *ExI*, the exocanthion inclination; *En-Na-En*, the angle of the endocanthions to nasion; *Pu-Na-Pu*, the angle of the pupils to nasion; *Ex-Na-Ex*, the angle of the exocanthions to nasion. *p ≤ 0.05; **p ≤ 0.001

The endocanthion-related measurements included En-En, En-Na, Pu-En, En-Na-En, and EnI. En-En significantly differed between the young and old groups (*p* < 0.001), but no significant differences were found between sexes in both the young and old groups (*p* = 0.175 and 0.683, respectively). However, women in the 80–90 subgroup showed differences with young and 70–80-year-old women (*p* < 0.001 and *p* = 0.045, respectively). En-Na distance did not significantly differ between the young and old groups (*p* = 0.253). However, sex differences existed in both the young and old groups (*p* < 0.001, respectively). Females in the 80–90-year subgroup showed differences with young females and those aged 60–70 for En-Na (*p* = 0.0031 and 0.0240, respectively). The Pu-En distance did not significantly differ between the young and old groups (*p* = 0.958). However, sex differences existed in both the young and old groups (*p* = 0.003 and 0.001, respectively). The En-Na-En was not significantly different between the young and old groups (*p* = 0.685). Also, no difference between sexes existed in both groups (*p* = 0.707 and 0.078, respectively). In contrast, male volunteers in the 60–70-year subgroup showed a difference with young males and females in the 60–70-year subgroup for En-Na-En (*p* = 0.047 and 0.035, respectively). The EnI was not significantly different between the young and old groups (*p* = 0.348). However, both the young and the old groups showed sex-related differences (*p* = 0.041 and 0.002, respectively). In addition, there were differences between females in the 60–70- and 80–90-year subgroups (*p* = 0.018).

Pupil center-related measurements included Pu–Pu, Pu-Na, and Pu-Na-Pu. No significant difference was found between the young and old groups for Pu–Pu (*p* = 0.054); however, differences between sexes were observed in the young group (*p* = 0.001). Females in the young group also differed from 80- to 90-year-old females (*p* = 0.035). There was no difference in Pu-Na between the young and old groups (*p* = 0.218), but differences were found between sexes in both groups (*p* < 0.001). There was no difference in Pu-Na-Pu between the young and old groups (*p* = 0.234), and no significant difference was found between sexes in the young group (*p* = 0.496) and between the young and old women (*p* = 0.247, 0.475, and 0.774 in each subgroup, respectively). However, the Pu-Na-Pu results for females and males in the old group were different *(p* = 0.031).

The exocanthion-related variables included Ex-Ex, Ex-Na, Pu-Ex, Ex-Na-Ex, and ExI. No significant differences were observed between the young and old groups for Ex-Ex (*p* = 0.114); however, a difference between sexes existed in both the young and old groups (*p* = 0.001 and 0.005, respectively). A difference in Ex-Na was observed between the young and old groups (*p* = 0.004). For females, the difference also existed between young and 70–80-year-old females (*p* = 0.009). There were sex-related differences in Pu-Ex in both the young and old groups (both *p* < 0.001). There were also significant differences in Pu-Ex between the young and old age groups (*p* < 0.001). Results of Pu-Ex for young females were significantly different from females aged 70–80 and 80–90 years (both *p* < 0.001). In contrast, Pu-Ex for males in the young group was significantly different from all old male subgroups (*p* = 0.002, 0.001, and < 0.001, respectively). A difference was found between old men and women, with a *p* value of 0.014. However, Pu-Ex was not significantly different between females and males in the young group (*p* = 0.196). Ex-Na-Ex was significantly different between the young and old groups (*p* = 0.003). However, the values for female and male volunteers in the old group were different (*p* = 0.021). Ex-Na-Ex in the young male group was significantly different between males aged 60–70 and 80–90 years (*p* = 0.012 and 0.017, respectively), while we found no difference between young and old females (*p* = 0.341, 0.275, and 0.336, respectively). ExI was significantly different between the young and old groups (*p* < 0.001). ExI in the young female group was significantly different compared to old females in all subgroups (*p* = 0.037, 0.003, and 0.001, respectively), while ExI in the young male group differed with 80–90-year-old women (*p* < 0.001). Males in the 80–90-year-old subgroup showed differences with those in the 70–80-year-old subgroup and females in the 80–90-year-old subgroup (*p* = 0.003 and 0.007, respectively).

The PFW, En-En, Ex-Na, Pu-Ex, PFI, EnI, and En-Na-En significantly changed (all *p* < 0.05). The pupil-related measurements (Pu–Pu, Pu-Na, and Pu-Na-Pu) did not significantly differ between the young and elderly groups. However, Pu–Pu and Pu-Na were significantly different between the sexes. The En-Na, Pu-En, En-Na-En, and EnI did not significantly differ between the young and old groups. However, significant differences existed between sexes in both the young and old groups. More apparent changes were observed after the age of 80 years in females. There was a significant difference in En-En between the young and old groups; the difference was greater in women, especially those over 80 years old. Among females, there were significant differences between the young and older group over 80 years old (*p* < 0.001), and significant differences existed between females in the 70–80 and 80–90 subgroups (*p* = 0.045). Ex-Na, Pu-Ex, Ex-Na-Ex, and ExI were significantly different between the young and old groups. In addition, significant differences existed between the sexes, mainly occurring after 70 years for females and after 80 years for males. The PFW distance was significantly different between the young and old groups and became more significant after the age of 70 years for females and 60 years for males. The PFW distance was also significantly different between the two sexes, which could also be observed in the young groups. In addition, the difference between the young and old groups for PFI became evident after 80 years of age for females and 70 for males. The PFI was significantly different between sexes, which also existed in the young groups.

## Discussion

Increasing attention has been paid to changes in periocular morphology in the last decade. However, the positional change of the endocanthion, pupil, and exocanthion during aging is still controversial [2, 4–6, 8]. Therefore, the positional changes of these landmarks were investigated in Caucasian populations of different ages and sexes in this study. Linear distance measurements were calculated in three-dimensional as well as two-dimensional photographs. Angle measurements were obtained from two-dimensional images to reduce the effect of redundant skin on measurements of older volunteers.

**Fig. 3:**
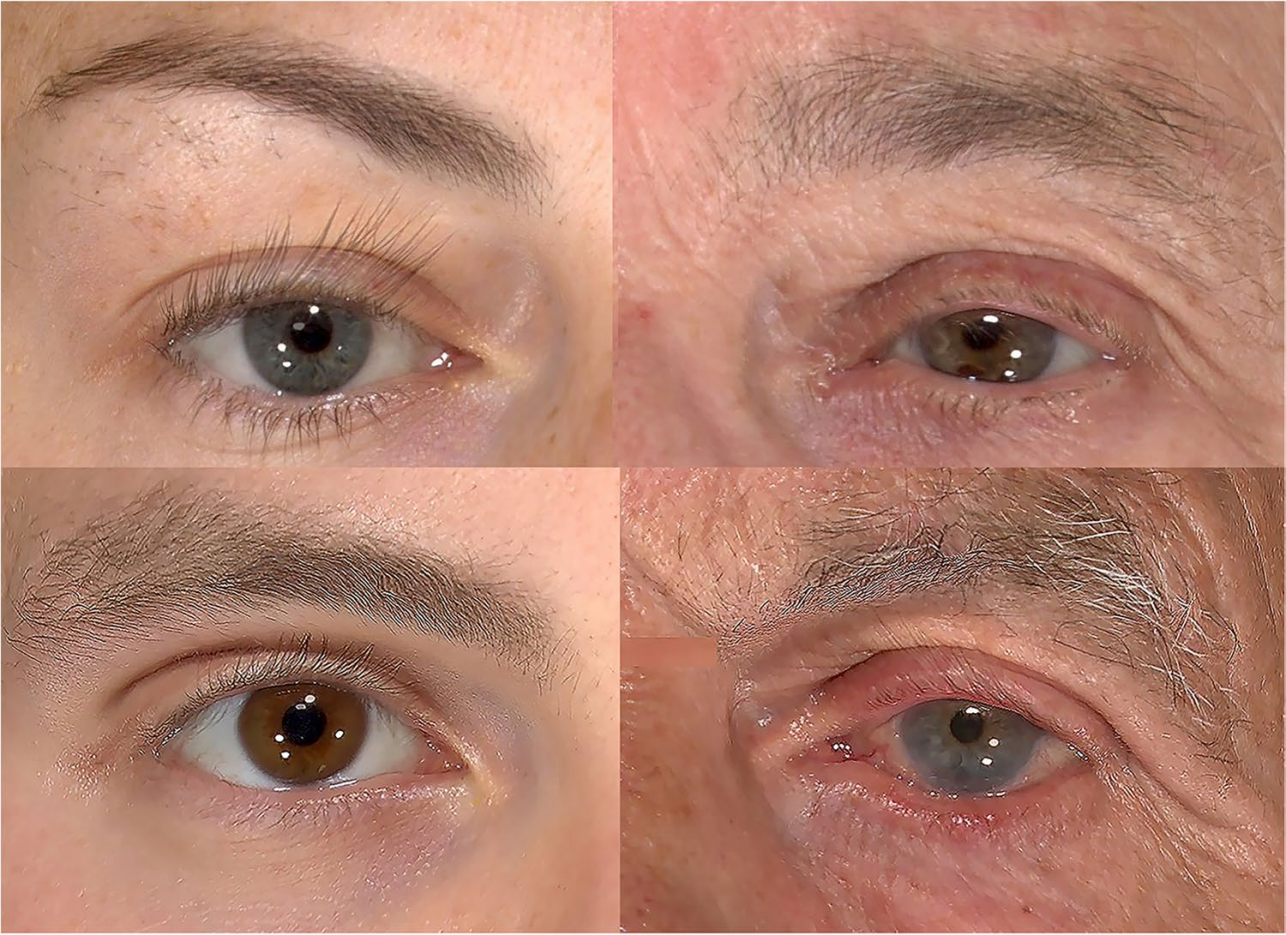
Periocular morphology changes in young and old people of different genders. Young female (above, left), old female (above, right), young male (below, left), old male (below, left)

From our study, we can draw the following conclusions. First, the change in pupil position is minimal relative to age compared with other landmarks, although it has a sex difference among Caucasian populations. Furthermore, the endocanthion was not significantly sex-related, but it tends to move temporally and inferiorly with age, and the exocanthion tends to shift to the nasal and inferior sides with age (as shown in Fig. 3). Also, both these changes become more significant after the age of 70 years in females and the age of 80 years in males.

Our results are supported by some existing literature. Price et al. [19] reported that exocanthion descent is a component of periorbital aging in females. Val Lambros et al. [10] reported that 74% of the old patients exhibited medial drift of the exocanthion. Bruneau et al. [4] also confirmed that the exocanthion tendon’s laxity might also induce variations with aging. Van den Bosch et al. [36] performed a cross-sectional cohort study of 320 healthy volunteers aged between 10 and 89 years. Their results showed that the width of the horizontal eyelid fissure was shortened between the middle-aged and old age groups. They also considered that aging does not affect the position of the eyeball, which is consistent with our results and those of Darcy et al. [5] who confirmed that the globe position does not change significantly with the bony orbit in the superoinferior axis. Park et al. [37] reported that the palpebral fissure slant gradually decreases with age in Korean females when they approach their 60 s. Rhee et al. [38] suggested that pupillary distance is nearly constant in each ethnicity and could be used as an ethnic characteristic, consistent with the study conducted by Jonathan et al. [11] and our results.

Our results also differ from some previous studies concerning periocular morphology change with age. Van den Bosch et al. [36] considered that aging does not affect the lateral canthus’ position. However, their study did not measure absolute distances, which may have affected the accuracy of results. Interestingly, Val Lambros et al. [10] compared photographs of the same patients and reported that the endocanthion did not move laterally. The reason for this contradictory conclusion could be that the two-dimensional photos they used do not always accurately reflect the actual location, especially for distance measurements. Moreover, the standardized photo collection is another weakness when compared with 3D photography [24, 25, 39].

The nasion is the most anterior point of the frontonasal suture that joins the nasal part of the frontal bone and the nasal bones [40]. As the most used landmark in maxillofacial surgery and otorhinolaryngology, it is less affected by age after puberty and is used frequently to establish a reference plane [41–48].

Val Lambros et al. [49] published a study in 2020, in which 594 patients were categorized into “young” and “old” groups by sex. Results of this study also showed that the horizontal eyelid distance of elderly volunteers was shortened by an average of 2.3 mm (*p* < 0.001) than young ones, and the lateral canthal angle moves medially. This conclusion was consistent with our research. However, his research only analyzes the horizontal changes of the eyelid fissure, and lacks the vertical direction, especially the changes in the movement trajectory relative to the nasion landmark.

Our results are also supported by some studies that focused on changes in orbital anatomy. Some studies indicate that due to weakened orbicularis muscle function, inferiorly directed tension is placed on the lower eyelid, which is attributed to slack palpebral skin and lateral tarsal ligaments and gravity, resulting in tendon attenuation and lower lid laxity [12]. Besides, some other studies [1, 50] have shown that the superomedial and inferolateral aspects of the orbital rim have a strong predisposition to resorption with aging as well as the orbital angle. They also showed that the mobility required for the function of the lateral orbital crow’s feet areas as well as the inferolateral orbital rim is structurally associated with a less ligamentous fixation of the soft tissues to the bone, which weakens the attachment of the muscles and ligaments to the bone in these areas. It is also reasonable to speculate that bone structure resorption could contribute to the facial ligament and muscle movement through the periosteum attachments. In addition, the ligament might move toward a more inferior inclined alignment as the ligament develops fatigue during aging [1]. Our results confirm that there is less movement of the endocanthion when compared with the exocanthion in older populations. In contrast, studies have shown that despite orbital bone absorption in the orbital rim, the eyeball position in the orbit can remain relatively stable [5], although some researchers have suggested enophthalmos due to orbital bone resorption [23].

Still, this study had some limitations. First, it was a cross-sectional population study based on comparisons among groups of individuals across different ages. Variations may affect the accuracy of results between individuals. Furthermore, although participants in the young and old groups are comparable, there were fewer volunteers in the 60–70 subgroup; thus, the results concerning this subgroup may not be as significant as those of the other subgroups. Nevertheless, in general, our sample size is adequate compared to other studies. Besides, the pupil position is often changed with eye movement, and obtaining the correct pupil position requires the operator’s repeated practice and good cooperation of the subject.

Additionally, we proposed the horizontal and vertical movements of the endocanthion and exocanthion with ages after a comprehensive analysis of linear distance in 3D photographs and angles in 2D photographs. However, we did not emphasize the sagittal changes of the pupil center, endocanthion, and exocanthion. On the one hand, the sagittal changes are impossible to measure for the pupil center and endocanthion blocked by the eyeball in lateral view. On the other hand, the sagittal movement has been incorporated in 3D positional changes, but it is not easy to separate it individually. To date, sagittal changes are often systematically hidden and ignored in 2D photography due to the lack of depth measurement, even though it might not be guaranteed that the above three landmarks would be stable in the sagittal direction with age. One of the study’s clinical significances is providing a more reliable and accurate reference plane for clinically eyebrow position-changing analysis, and we hope that this article might provide some guidance for 2D photography techniques. It is also hoped that sagittal changes can be better elucidated in the future as technology develops.

Nevertheless, our study provides researchers with further important information. First, the pupil is preferred over the endocanthion or exocanthion to establish the reference plane to describe periocular changes. Second, linear distance measurements using three-dimensional photographs are advantageous, while two-dimensional photographs are preferable for angular measurements.

In summary, our results demonstrated that the pupil is more stable during the aging process and is preferred to establish reference planes to describe the periocular aging changes. The exocanthion moves more dramatically than the endocanthion; both move inferiorly and toward the midline of the pupil with age. This process occurs earlier in females than in males.
